# Flexible Carbon Nanotube Modified Separator for High-Performance Lithium-Sulfur Batteries

**DOI:** 10.3390/nano7080196

**Published:** 2017-07-26

**Authors:** Bin Liu, Xiaomeng Wu, Shan Wang, Zhen Tang, Quanling Yang, Guo-Hua Hu, Chuanxi Xiong

**Affiliations:** 1 State Key Laboratory of Advanced Technology for Materials Synthesis and Processing, School of Materials Science and Engineering, Wuhan University of Technology, Wuhan 430070, China; kingingben@outlook.com (B.L.); wxm07156@163.com(X.W.); zhentang@whut.edu.cn (Z.T.); yangql@whut.edu.cn (Q.Y.); hufrance@hotmail.com (G.-H.H.); 2 Laboratory of Reactions and Process Engineering (LRGP, CNRS UMR 7274), CNRS-University of Lorraine, 1rue Grandville, BP 20451, 54001 Nancy, France

**Keywords:** Lithium-sulfur batteries, carbon nanotubes, separators, shuttle effect

## Abstract

Lithium-sulfur (Li-S) batteries have become promising candidates for electrical energy storage systems due to their high theoretical specific energy density, low cost and environmental friendliness. However, there are some technical obstacles of lithium-sulfur batteries to be addressed, such as the shuttle effect of polysulfides. Here, we introduced organically modified carbon nanotubes (CNTs) as a coating layer for the separator to optimize structure and enhance the performance of the Li-S battery. The results showed that the cell with a CNTs-coated separator exhibited an excellent cycling performance. Compared to the blank separator, the initial discharge capacity and the capacity after 100 cycles for the CNTs-coated separator was increased by 115% and 161%, respectively. Besides, according to the rate capability test cycling from 0.1C to 2C, the battery with a CNTs-coated separator still released a capacity amounting to 90.2% of the initial capacity, when the current density returned back to 0.1C. It is believed that the organically modified CNTs coating effectively suppresses the shuttle effect during the cycling. The employment of a CNTs-coated separator provides a promising approach for high-performance lithium-sulfur batteries.

## 1. Introduction

Nowadays, rechargeable lithium batteries have been widely used in portable electronics, electric vehicles and various energy storage devices. Lithium-sulfur (Li-S) batteries have been considered to be one of the most promising choices for next generation, high-energy rechargeable batteries due to their high theoretical energy density. For lithium-ion batteries with graphite as negative electrodes, the theoretical energy densities are typically limited to around 400 Wh/kg or 1400 Wh/L [1,2], while the theoretical energy densities of lithium-sulfur batteries are calculated to be 2500 Wh/kg or 2800 Wh/L [3,4]. Also sulfur offers a theoretical capacity of 1675 mAh/g, which is higher than those of transition-metal oxide electrode materials by an order of magnitude [5]. Additionally, the merits of abundant reserves, low cost and environmental friendliness of sulfur are greatly in favor of large-scale commercialization of Li-S batteries.

In spite of these advantages, there are still great challenges limiting the practical application of Li-S batteries. On one hand, there is low utilization of the active materials in the cathode since both sulfur and its discharge product Li_2_S are electronically and ionically insulating. On the other hand, due to the soluble characteristics and thus the shuttle behavior of the polysulfide intermediates (Li_2_S*_x_*, 4 ≤ *x* ≤ 8), which are generated during the charge/discharge process, irreversible capacity loss is commonly unavoidable. This consequently leads to low coulombic efficiency and poor cycle life for Li-S batteries [6].

To address these key issues, great efforts have been devoted in the past decades mainly toward improving the electrochemical performance of the sulfur cathode. A variety of carbon materials such as carbon nanofibers [7], graphene [8,9] and porous carbon [10] have been employed to fabricate sulfur/carbon composite cathode to enhance the conductivity of sulfur as well as to suppress the diffusion of dissolved polysulfides out of the cathode. Also conductive polymers have been adopted and have demonstrated their role in improving the electrochemical performance of the sulfur cathode [11]. However, the enhanced electrochemical performance is usually compromised by the lower sulfur loading mass in the cathode, which greatly reduces the practical energy density of Li-S batteries. Alternatively, a promising strategy based on modifying the separator with conductive materials (e.g., Super P, Ketjen Black) has been recently proposed and demonstrated its great effectiveness on improving the rate capability and cycle life of Li-S batteries [12,13]. As an essential part of batteries, separators are placed between anode and cathode to prevent physical contact of the electrodes during the free ion transfer process. The properties of separators such as composition, porosity and wettability can affect the energy density, cycle life and safety of the battery [14]. It was claimed that the conductive coating or layer on separator could facilitate electron or ion transport, enhance electrolyte uptake, and inhibit the shuttle effect of polysulfides [15].

Owing to their outstanding electrical conductivity, one-dimensional carbon nanotubes (CNTs) have attracted considerable interest in the field of the lithium sulfur battery, especially in terms of the electrodes [16,17]. Few studies have involved coating the separator with CNTs due to their entanglement, poor solubility and dispersion in solvents. Chung et al. fabricated CNTs-coated separator by a simple vacuum filtration technique, and the results showed that Li-S battery with the modified separator possessed high discharge capacity, excellent rate performance and long cycle life [18]. However, low efficiency and safety problems (i.e., separator rupture) may be involved in the vacuum filtration process. Therefore, it is highly desirable to design a facile but efficient route to apply the CNTs coating on the commercial separator.

In this work, flexible carbon nanotubes were first prepared by oxidation followed by a grafting route. Then the modified CNTs were spin coated onto the surface of the commercial separator, and assembled into Li-S battery. The electrochemical performance of lithium-sulfur batteries was comprehensively studied. The hydrophilic groups and reticular structure in the organically modified CNTs coating layer effectively suppressed the shuttle effect by trapping dissolved long-chain lithium polysulfides(Li_2_S*_x_*, *x* = 4, 6, 8). The introduction of organically modified CNTs enhanced the thermal shrinkage property of the separator, which is beneficial for its practical application. Besides, the conductive nature of CNTs also benefited the decrease of the internal resistance. To sum up, modified CNTs-coated separators are promising for high performance lithium-sulfur batteries. 

## 2. Results and Discussion

### 2.1. Morphology and Structural Characterization

**Figure 1:**
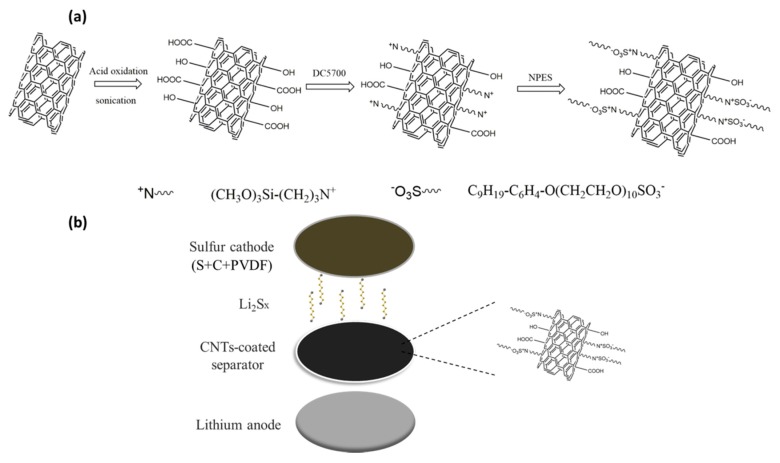
Schematic illustration of (**a**) organic modification process of carbon nanotubes and (**b**) cell configuration of lithium-sulfur (Li-S) batteries.

As shown in Figure 1a, the organic modification process of carbon nanotubes consisted of three successive steps. Carbon nanotubes were first oxidized by a mixed, concentrated acid of H_2_SO_4_ and HNO_3_, resulting in a great amount of hydrophilic groups including carboxyl and hydroxyl on the surface of carbon nanotubes. An organic silane ((CH_3_O)_3_Si(CH_2_)_3_N^+^(CH_3_)_2_(C_18_H_37_)Cl^−^) (DC 5700) was adopted to couple and a long-chain protonic acid containing a polyethylene glycol segment (C_9_H_19_-C_6_H_4_-O(CH_2_CH_2_O)_10_SO_3_^−^Na^+^) (NPES) was applied to exchange ion successively [19,20]. The as-obtained products were spin coated onto the commercial Celgard 2325 separator and the coated separators were assembled into lithium-sulfur batteries. As shown in Figure 1b, the coated separators were designed to trap and prevent lithium polysulfides from crossing the separators, which would effectively suppress the shuttle effect.

**Figure 2:**
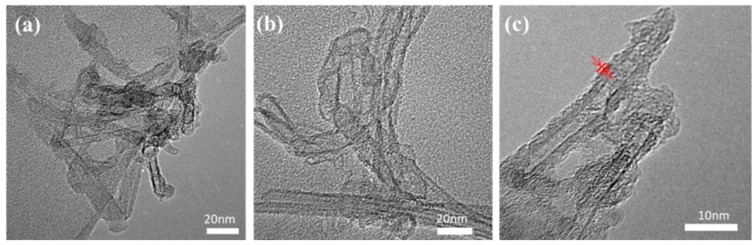
TEM images of (**a**) original carbon nanotubes (CNTs), (**b**) oxidized CNTs and (**c**) organically modified CNTs.

Typical transmission electron microscope (TEM) images of the original and oxidized CNTs are shown in Figure 2a,b. Comparing to the original CNTs, it is found that less entanglements are present for the oxidized CNTs. This could be due to the successful introduction of some hydrophilic groups such as hydroxyl and carboxyl in the oxidized process, consequently leading to an improved dispersion of CNTs in water. Figure 2c shows the TEM of the synthesized CNTs. It can be seen that the CNTs are surrounded by a shell structure with a thickness of about 1 nm, which is the organic shell formed by DC 5700 and NPES long-chain molecules grafted on the CNTs surface. After graft modification, the entanglement of CNTs reduced greatly. The CNTs could be easily dispersed in some solvents such as chloroform, and form a homogeneous slurry state, which facilitates the coating technique.

**Figure 3:**
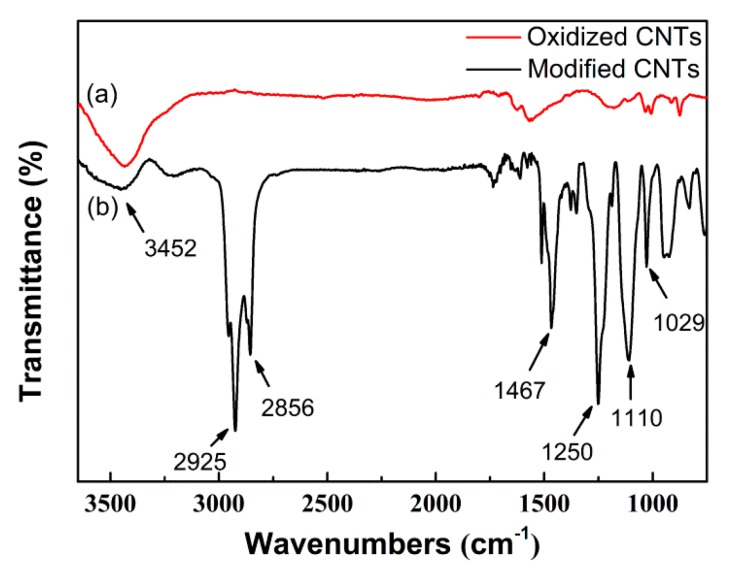
FTIR spectra of (**a**) oxidized CNTs and (**b**) organically modified CNTs.

Figure 3 compares the Fourier transform infrared (FTIR) spectra of the oxidized and organically modified CNTs. Some additional characteristic peaks can be distinctively observed in the spectrum of the organically modified CNTs. Specifically, characteristic peaks around 2925 cm^−1^ and 2856 cm^−1^ corresponded to the stretching vibration of methyl and methylene, while the peak at 1467 cm^−1^ represented the bending vibration of methylene. The peak at 1251 cm^−1^ was characteristic of the –SO_3_ group and the peaks of 1170 cm^−1^ and 1029 cm^−1^ were attributed to the Si–O–Si vibration. Besides, it was found that the vibration strength of the hydroxyl peak at 3450 cm^−1^ reduced for the organically modified CNTs, indicating that the amount of hydroxyl groups decreased due to the reaction between the CNTs and the organic silane DC 5700. Combined with the chemical structure of the DC 5700 and NPES, it was reasonably deduced that the organic modifiers have been successfully grafted onto the walls of CNTs in this study.

**Figure 4:**
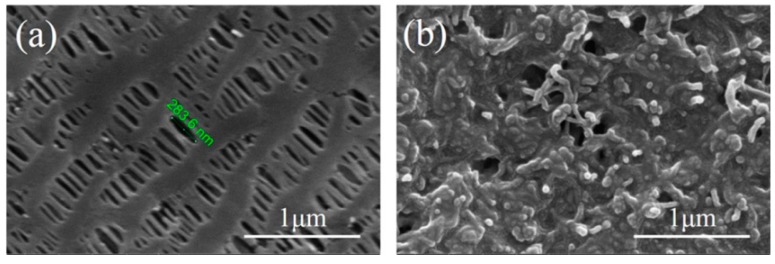
SEM images of (**a**) blank separator and (**b**) CNTs-coated separator.

Figure 4a,b show the scanning electron microscope (SEM) images of the blank and CNTs-coated separators, respectively. Typically, the blank separator displayed uniform slit-like micropores with a length of about 280 nm and a width of about 70 nm. It is known that these nanosized micropores could provide a convenient channel for transporting the free lithium ion. Unfortunately, it was also said that this type of micropore allowed the shuttle of the dissolved polysulfides during the charge/discharge process, which consequently deteriorated the rate performance and shortened the cycle life [21]. While for the CNTs-coated separator, it is clear that the tubular carbon materials were uniformly adhered to the surface of the separator and the original porous structure was entirely covered. The long CNT tubes intertwined with each other and constructed a large number of nanosized pore structure in the coating layer. According to previous reports, this structure could facilitate the storage of the polysulfide intermediates [18].

**Figure 5:**
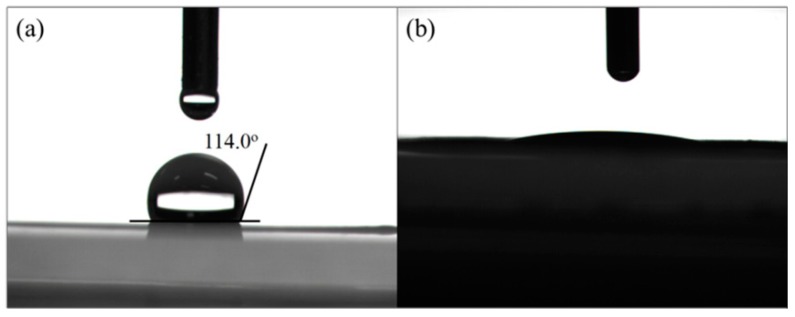
Water contact angles of (**a**) blank separator and (**b**) CNTs-coated separator.

Essentially, the surface chemistry property of separator plays an important role in controlling the diffusion of polysulfides. Considering the water contact angle (WCA) can reflect the polarity of the separators, WCA measurements were carried out as shown in Figure 5. The WCA of the blank separator was approximately 114.0° (Figure 5a), which associated well with the hydrophobic nature of the polypropylene-based separator. In contrast, the water droplet quickly spread as soon as it contacted with the surface of the CNTs-coated separator, and the WCA measured at 10 s was only about 15°, suggesting a good hydrophilicity. The result was in consistence with the previous FTIR analysis, indicating the presence of long-chain organic modifier and abundant hydrophilic groups including hydroxyl and carboxyl groups on the walls of the CNTs. And it is known that hydrophilicity of separator would benefit for the suppression of shuttle effect by absorbing polysulfides [8,22].

Considering the main function of separators in preventing physical contact of anode and cathode, separators should be stable at high temperature, which is common in practical application. In order to assess the thermal stability of the separators at high temperatures, the separators were treated at different temperatures from 120 °C to 155 °C for 30 min to calculate the shrinkage percentages as shown in Table 1. It is clear that the thermal shrinkage of the CNTs-coated separator was less than that of blank separator under the same thermal circumstance, suggesting an improved stability upon the CNTs modification. Take 150 °C as an example, the shrinkage of the CNTs-coated separator is 25%, which is much lower than that of blank separator (38.3%). It was suggested that the CNTs skeleton could prevent the closure of micropores and thus ensure the stability of separators at high temperature.

### 2.2. Electrochemical Performance

**Figure 6:**
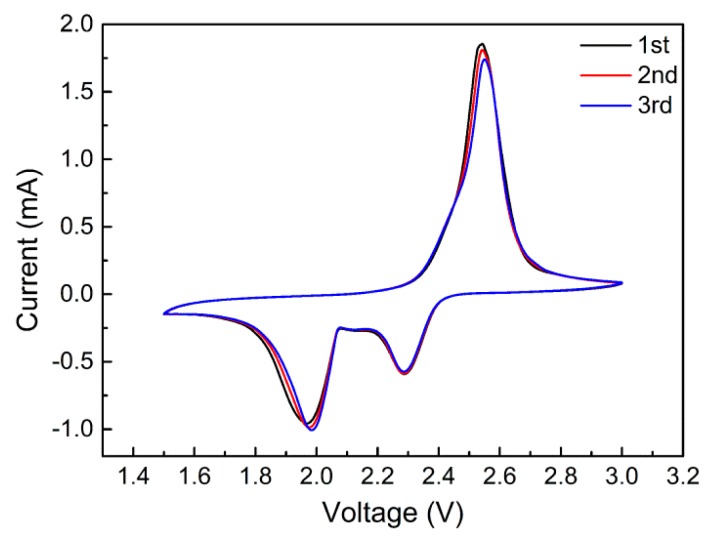
Cyclic voltammograms of the cells with CNTs-coated separator.

The cyclic voltammetry was applied to clarify the mechanism of lithium-sulfur batteries as shown in Figure 6. The cyclic voltammogram of the CNTs-coated separator owned three distinct peaks including two reduction peaks and one oxidation peak. Compared to the cell with commercial separator (not shown here), no additional peaks were observed for the cell loaded with the CNTs-coated separator, indicating that the CNTs coating layer did not participate in the redox reaction during the charge/discharge process. Generally, the reduction peaks around 2.3 V and 2.0 V corresponded to the conversion from S_8_ to long-chain polysulfides (Li_2_S*_x_*, 4 ≤ *x* ≤ 8) and the formation of Li_2_S_2_/Li_2_S, respectively, while the oxidation peak centered around 2.5 V suggested the typical elemental sulfur converting process [23,24,25]. Besides, it should be noted that the cyclic voltammograms in Figure 6 almost coincided with each other in the first three cycles, indicating a high reversibility of electrochemical reaction for the battery with the CNTs-coated separator, which could be ascribed to the suppression of shuttle effect and stability of the battery.

**Figure 7:**
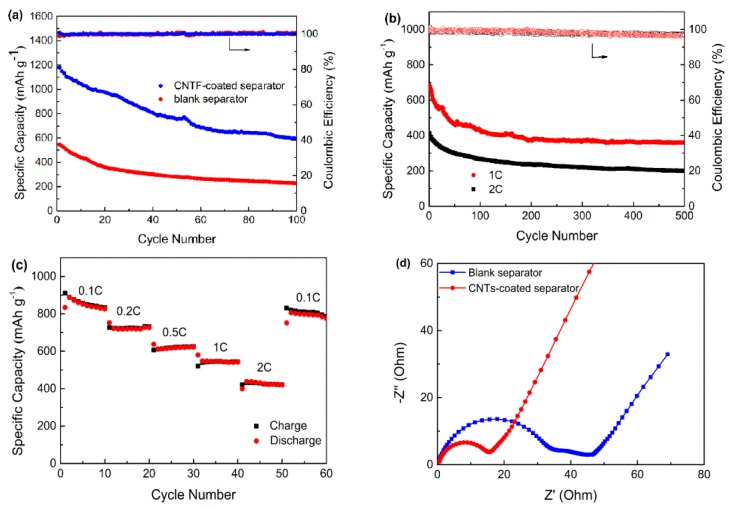
(**a**) Cycling performance of the blank separator and CNTs-coated separator at 0.1C; (**b**) Cycling performance of the CNTs-coated separator at the rates of 1C and 2C; (**c**) Rate performance of the Li-S batteries with the CNTs-coated separator; (**d**) EIS data of the cells with the blank separator and CNTs-coated separator.

Figure 7a compares the discharge capacity of the Li-S batteries with the blank separator and CNTs-coated separator. It was found that the cell with the CNTs-coated separator delivered an initial discharge capacity as high as 1179 mAh/g at 0.1C while the cell with the blank separator only delivered an initial value of 547 mAh/g. In addition, the discharge capacity of the CNTs-coated and blank separators remained at 595 mAh/g and 228 mAh/g after 100 cycles, suggesting the capacity retention rates of 50.6% and 41.7%, respectively. This suggested that modification of the separator with the CNTs coating improved not only the initial discharge capacity but also the capacity retention rate. The improvement should be ascribed to the CNTs coating, which was capable of trapping long-chain polysulfides during the cycling.

The cycling performance of the Li-S battery with the CNTs-coated separator at high rates was further analyzed as shown in Figure 7b. The reversible capacity of the battery dropped from 684 mAh/g to 361 mAh/g at 1C in 500 cycles, with a coulombic efficiency of 96.7% on average and a capacity retention rate of 52.8%. When the rate increased to 2C, the initial discharge capacity of the battery was 414 mAh/g and remained 200 mAh/g after 500 cycles, suggesting an average capacity decay rate of only 0.1% per cycle. The coulombic efficiency also kept as high as 96.8%. The result is encouraging since it suggested the CNTs coating in the work could maintain the structural stability of the battery. That is to say, it would not collapse, even as the charge and discharge occurs rapidly, consequently leading to a long cycle life of the Li-S battery at high current density. Figure 7c presents the rate performance of the Li-S batteries with the CNTs-coated separator. With an initial value of 887 mAh/g at 0.1C, the capacity remained around 720, 620, 545 and 430 mAh/g at 0.2C, 0.5C, 1C and 2C in 10 cycles, respectively, and returned back to 800 mAh/g when the rate was 0.1C. The excellent reversibility of the capacity as high as 90.2% supported strongly again that the Li-S battery with CNTs-coated separator was highly stable and reversible. 

Electrochemical impedance spectra (EIS) of the battery with and without CNTs-coated separator are shown in Figure 7d. The Nyquist plots for this battery consisted of a semicircle in high frequency region, another in medium-to-low frequency region and an oblique line in the low-frequency region, which represented the resistance from a passivation film (*R_t_*), the charge-transfer resistance (*R_ct_*) and Warburg impedance, respectively [26,27]. According to the previous reports, the diameter size of the semicircle and slope size of the oblique line represented the value of impedance. It is clear that *R_ct_* and Warburg impedance are similar to each other for the different battery, while the *R_t_* from a passivation film of the battery with CNTs-coated separator is less than that of the battery with blank separator. The results indicated that CNTs coating could significantly reduce the *R_t_*, due to the conductive nature of CNTs and the enhanced electrolyte absorption capability. Similar to the previous study by other researchers, the enhanced electrical conductivity of the battery with CNTs coated separator led to a better rate capability and a high reversible capacity than that with blank separator [28]. The result was consistent with the preceding enhanced cycling performance.

## 3. Materials and Methods

### 3.1. Materials

Multi-walled carbon nanotubes (CNTs) were provided by Chengdu Organic Chemicals Co., Ltd. (Chengdu, Sichuan, China) Ethanol, chloroform, concentrated sulfuric acid (H_2_SO_4_, 98%), and concentrated nitric acid (HNO_3_, 65%) were purchased from Sinopharm Chemical Reagent Co., Ltd. (Shanghai, China). Polysiloxane quaternary ammonium salt DC5700 [(CH_3_O)_3_Si(CH_2_)_3_N^+^(CH_3_)_2_(C_18_H_37_)Cl^−^] in methanol (40%) was provided by Gelest (Morrisville, PA, USA). Sulfonate salt NPES [C_9_H_19_C_6_H_4_O(CH_2_CH_2_O)_10_SO_3_^−^K^+^] was obtained from Aldrich (Shanghai, China). Sublimed sulfur was purchased from Sinopharm Chemical Reagent Co., Ltd. (Shanghai, China). Celgard 2325 from Celgard, LLC (Charlotte, NC, USA) was adopted as separator. Bis(trifluoromethane) sulfonamide lithium(LiTFSI), lithium nitrate (LiNO_3_, 99.99% trace metals basis), *n*-butyl alcohol (99%), *N*-methyl-2-pyrrolidone (NMP, 99%), 1,3-dioxolane (DOL) and 1,2-dimethoxyethane (DME) were obtained from Aladdin (Shanghai, China).

### 3.2. Preparation of CNTs-Coated Separator

Typically, CNTs were first oxidized in a mixture of concentrated H_2_SO_4_ and HNO_3_ with a volume ratio of 3:1 at 50 °C for 3 h. The suspension was then diluted with deionized water, followed by a centrifugation to remove the supernatant acid. The remaining solids were washed repeated with deionized water until the pH value reached about 3. DC5700 was then added into the aqueous solution of oxidized CNTs to render them cationic. The mixture was sonicated for 2 h, washed with water and anhydrous ethanol successively, and dried at 70 °C for 6 h. Subsequently, the resultants and a certain proportion of NPES were mixed in the chloroform solution. The mixture was stirred for 5 h at room temperature to ensure a full reaction, followed by a dialysis for 24 h in the deionized water bath to completely remove the KCl salt and excess sulfonate salt. Finally, the product was vacuum-dried at 70 °C for 24 h to obtain the modified CNTs.

An appropriate amount of CNTs was dissolved in the chloroform to prepare the CNTs slurry. Then a small amount of CNTs slurry was spin-coated onto the surface of the virgin separator. Before the coating operation, the virgin separator was cleaned with anhydrous ethanol in an ultrasonic bath to remove any contamination. The coated separator was then dried in a vacuum oven at 50 °C for 24 h.

### 3.3. Characterizations

TEM images were made on a JEM 2100F field emission transmission electron microscope (Japan Electronics Co., Ltd., Tokyo, Japan). FTIR analysis was carried out using a Nicolet 6700 Fourier transform infrared spectrometer (Thermo Fisher Scientific Inc., Waltham, WA, USA). The surface morphologies of the virgin and coated separators were observed using a field emission scanning electron microscope (SEM, QUANTA FEG 450, FEI, Hillsboro, OR, USA). The water contact angles of the separators were measured by a JC 2000C contact angle measuring instrument (Powereach, Shanghai, China). The thermal shrinkage ratio *S* was calculated according to the expression $S=\left(L_{0}-L\right)/L_{0}\times 100\%$, where *L*_0_ and *L* are the lengths of the separators before and after the thermal treatment, respectively.

The CR2032 coin cells were assembled in an argon-filled glove box to evaluate the electrochemical performance of the virgin and CNTs-coated separator. The electrolyte was made up of 1 M bis(triuoromethanesulfonyl)imide (LiTFSI) in dioxolane/dimethoxyethane solution (DOL/DME, *V*/*V* = 1:1) with 1 wt % LiNO_3_ additive. The cathode was prepared by mixing elemental sulfur, acetylene black and poly(vinylidene fluoride) with a weight ratio of 3:2:1 in *N*-methyl-2-pyrrolidinone. The slurry was coated on the aluminum foil and dried under vacuum to form the working cathode. Lithium metal foil was adopted as the anode. Cycling performance of the batteries was analyzed using a cell testing system (LAND CT2001A, Wuhan LAND electronics, Wuhan, China). Both the cyclic voltammetry curves (CV) and AC impedance of the battery were measured using a CHI 660E electrochemical workstation (CH Instruments, Shanghai, China). The voltage range was controlled in 1.5~3 V and the scanning rate was 0.1 mV/s. For the AC impedance measurement, the scanning frequency range was controlled from 0.01 to 100 kHz, with an AC potential amplitude of 5 mV at the open-circuit potential.

## 4. Conclusions

In summary, we proposed a novel and effective method for the modification of separators in lithium-sulfur batteries. Grafted long-chain molecules and tube-like CNTs enable the formation of micropores in CNTs, which could accommodate polysulfides. The hydrophilic groups help trapping polysulfides to suppress shuttle effect. The organically modified CNTs coating also facilitate the battery with a high temperature stability. In addition, the conductive nature of CNTs also contributes to a decrease in internal resistance. The performance of the battery is improved significantly after adopting our method, indicating new avenues for practical lithium-sulfur batteries.

## Figures and Tables

**Table 1:** Thermal shrinkage of blank separator and CNTs-coated separator.

Separator	Shrinkage of Separator (%)				
	120 °C	130 °C	140 °C	150 °C	155 °C
Blank	6.7	10.3	21.7	38.3	43.0
CNTs-coated	5.0	9.3	16.0	25.0	35.0
